# Monoclonal antibodies in cervical malignancy-related HPV

**DOI:** 10.3389/fonc.2022.904790

**Published:** 2022-10-06

**Authors:** Parisa Shiri Aghbash, Nima Hemmat, Hamidreza Fathi, Hossein Bannazadeh Baghi

**Affiliations:** ^1^ Immunology Research Center, Tabriz University of Medical Sciences, Tabriz, Iran; ^2^ Infectious and Tropical Diseases Research Center, Tabriz University of Medical Sciences, Tabriz, Iran; ^3^ Department of Virology, Faculty of Medicine, Tabriz University of Medical Sciences, Tabriz, Iran; ^4^ Drug Applied Research Centre, Tabriz University of Medical Sciences, Tabriz, Iran; ^5^ Network of Immunity in Infection, Malignancy and Autoimmunity (NIIMA), Universal Scientific Education and Research Network (USERN), Tabriz, Iran

**Keywords:** anti-EGFR, anti-VEGF, cervical neoplasm, immune checkpoint inhibitors, monoclonal antibody

## Abstract

Despite many efforts to treat HPV infection, cervical cancer survival is still poor for several reasons, including resistance to chemotherapy and relapse. Numerous treatments such as surgery, radiation therapy, immune cell-based therapies, siRNA combined with various drugs, and immunotherapy are being studied and performed to provide the best treatment. Depending on the stage and size of the tumor, methods such as radical hysterectomy, pelvic lymphadenectomy, or chemotherapy can be utilized to treat cervical cancer. While accepted, these treatments lead to interruptions in cellular pathways and immune system homeostasis. In addition to a low survival rate, cervical neoplasm incidence has been rising significantly. However, new strategies have been proposed to increase patient survival while reducing the toxicity of chemotherapy, including targeted therapy and monoclonal antibodies. In this article, we discuss the types and potential therapeutic roles of monoclonal antibodies in cervical cancer.

## 1. Introduction

The World Health Organization (WHO) estimated 570,000 new patients and 311,000 cervical cancer deaths globally in 2018, making it the second most common malignancy among women, with developing regions bearing more than 85% of the global burden (1). Persistent infection with the human papillomavirus (HPV) can lead to cervical cancer and other related malignancies. A study in 2008 predicted that society would witness an 80% increase in new cases over the next few years (2, 3). In general, HPV infection occurs in four stages: 1. HPV transmission, 2. viral stability, 3. continuous progression of cell infection, and 4. precancerous lesions to invasive cervical lesions (4). In this respect, one of the high-risk agents that lead to persistent HPV infection is suppressing the immune system (5). Following HPV infection, cervical cancer development, and secretion of viral antigens (6), immunotherapy for cervical cancer has expanded in popularity Given the immune system’s ability to detect and eliminate infected and tumoral cells, these cells have the potential to evade detection and removal. For instance, disruption of major histocompatibility complex (MHC) I and other innate immune system components leads to the stability of infected cells and cervical cancer progression. Furthermore, several HPV proteins, such as E1 and E2 and oncoproteins E5, E6, and E7, promote the secretion of immunosuppressive cytokines which suppress the immune response (7). In this way, upon E6 and E7 inactivation, oncogenes’ processes are not accrued; hence, these oncogenes might be an effective target for therapy (8).

**Figure 1:**
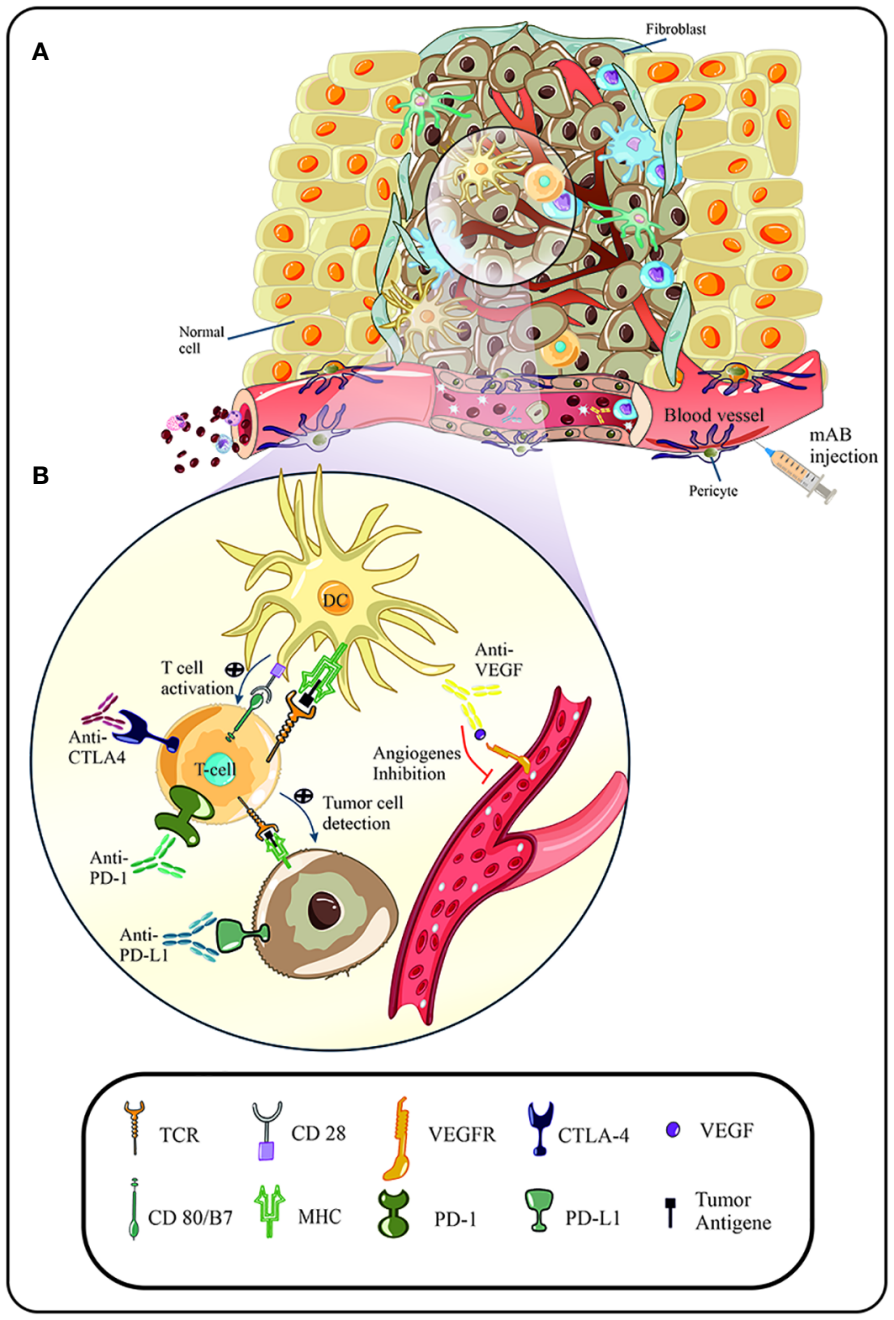
**(A)** Suitable conditions of the tumor microenvironment lead to increased uptake and penetration of immune cells in the malignancy area. Due to the escape mechanisms of human papillomavirus (HPV), immune cells, such as T cells, are unable to detect tumor cells, and become anergic following cellular pathways and signals. In addition, increased expression of immune checkpoints (CTLA-4, PD-1) and cellular factors (VEGF) are directly related to the rate of angiogenesis and progression of the malignancy. **(B)** Following immunotherapy, such as the use of immune checkpoint inhibitors, blockade of immune checkpoints and subsequent activation of signaling pathways of immune cell activity is observed. In addition, anti-VEGF agent inhibit the binding of VEGF to its related receptor and prevent increased angiogenesis and progression of the malignancy.

During HPV infection, increased expression of programmed death-ligand-1 (PD-L1) and cytotoxic T-lymphocyte-associated protein-4 (CTLA-4) on the surface of cancer cells leads to escape from the immune system and the progression of malignancy (9). PD-L1 is not expressed in normal cervical cells and benign cervical lesions (10–12). Moreover, immunomodulatory therapies such as PD-1/PD-L1 inhibitors, CTLA-4, 4-1BB, and other cellular pathways including vascular endothelial growth factor (VEGF) and epidermal growth factor receptor (EGFR) are studied in clinical trials (13) (Monoclonal antibodies related to each of the mentioned items are listed as a table in a **Supplementary File**). Despite the improvements in our understanding of HPV infection and subsequent cervical cancer processes, no particular therapy has been recommended in clinical trials yet (14–16). For example, surgery, radiation, and hormone chemotherapy are presently utilized for cervical cancer treatment, even though they have some side effects. Nevertheless, immunotherapy has been indicated as a promising approach in treating cervical cancer. This novel therapeutic strategy for HPV-related cervical cancer is very effective, specific, and non-toxic (7). In this regard, over several decades, various forms of HPV protein antibodies, either polyclonal or monoclonal antibodies, have been designated against multiple types of HPV proteins or cancer cells’ surface proteins and stimulated pathways during the infection to improve cervical cancer diagnosis and treatment (17–19). For example, monoclonal antibodies have been commonly used to detect HPV16 virus-like particles (VLP) epitopes (20). The immunological methods through their high affinity, specificity, and biocompatibility have been proposed to advance diagnostic and therapeutic strategies for HPV induced-cancers (21). Carcinogenesis is a complicated process that begins with precancerous lesions and progresses to cancer, hence, requiring a novel approach to prevention, diagnosis, and therapy (5). This manuscript reviews a variety of approved monoclonal antibodies in the experimental phase for treating cervical cancer, preventing its progression in early stages, and reducing the side effects of other therapies (**Figure 1**).

## 2. Human papillomavirus and cervical cancer

Emerging molecular tests have revealed papillomavirus RNA and DNA traces in various areas of the genital skin, genital warts, and biopsies obtained from cervical cancer over the last few decades. Subsequently, studies by Zur Hausen H et al. revealed several links between papillomavirus and cervical cancer (22, 23). To date, more than 200 different types of human papillomavirus have been identified that cause various lesions in human skin and mucous membranes. However, cervical cancers and other malignancies are caused by several different types of high-risk HPV. Among the high-risk types, HPV16 and HPV18 are most commonly associated with cervical cancer and other malignancies in the vagina, penis, anus, and oropharynx (24). The most common histopathological subtypes of all cervical cancers include squamous cell carcinoma (SCC) (70%) and adenocarcinoma (25%) (25). The virus has two protein-coding regions that encode eight proteins: the end region of the genome encodes two capsid proteins, and the beginning region of the genome encodes six proteins involved in proliferation and carcinogenesis. E1, as a helicase enzyme, plays a major role in the replication of the viral genome and, together with E2, plays an effective role in regulating the transcription of viral genes, especially E6/E7 oncoproteins, and the proliferation of the viral genome during the intracellular viral replication cycle (26). As mentioned above, the main oncogenes of HPV, which play a key role in viral replication and spread of the infection, are E6 and E7, which integrate into the host genome during infection and are highly expressed in malignancies. E5 is also an oncogene that is not expected to integrate into the host genome (27–30). Some studies have also shown that the amplification of the viral genome, the proliferation of the E6/E7 oncogenes, and cell cycle arrest during the G2/M phases are associated with HPV E4, presumably due to the interaction of E4 with both E2 and mitogen-activated protein kinase (MAPK) activation (31, 32). Moreover, the E5 gene participates in immune response inhibition, carcinoma progression, and in cooperation with E7, it can be involved in the progression of cancer in HPV infection, especially HPV16 (33, 34). Despite the inhibition of retinoblastoma protein (pRb) and p53, by E6/E7 oncoproteins in cervical cancer, there is conflicting data on the carcinogenic role of E2/E4/E5. A study by Shuling Ren et al. showed that these proteins also play a significant role in primary oral keratinocytes cancer in mice (cK5-rtTA transgenic mice) (35, 36). HPV structural proteins, including L1 (primary capsid protein) and L2 (partial capsid protein), are encoded by the terminal region. These proteins also contribute to cervical cancer progression. Also, during carcinogenesis, HPV-infected cells enhance the pathogenesis process or disrupt its identification by the immune system through altering the cell’s natural signals. Several transduction signals, including wingless/int (Wnt/β-catenin), Phosphatidylinositol-3-Kinase/Protein kinase B (PI3K/Akt), extracellular signal-regulated kinase (ERK)/MAPK, and PD-1/PD-L1 involved in cell proliferation and differentiation, are disrupted in HPV-infected cells (37). These pathways can be tempting options for treating HPV-related cancers (26, 38). A previous study found a direct correlation between PD-L1 and PD-1 expression levels, cervical intraepithelial neoplasia (CIN) grade, and HPV positivity rate, implying that the PD-1/PD-L1 pathway could impair cervical immunity in CIN-related high-risk human papillomavirus (hr-HPV) (9). For example, in the study of CIN with different degrees and levels of HPV genes, a close relationship has been observed between increasing the presence of viral genes and increasing CIN with the expression levels of each of the PD-L1 and PD-1 genes, which cause immunosuppression in cervical cancer areas (9).

## 3. Immune response against cervical cancer

During HPV infection, the innate immune response is the first line of defense and a robust inflammatory response is initiated to protect against infected cells. Immune cells such as dendritic cells (DC), langerhans cells (LC), natural killer cells (NK), and natural killer T cells (NKT) drive the immune response, and promote cytokine-dependent inflammatory secretion and penetration into the infected area during the early immune response (7). It has also been shown that the production of type I IFNs activates acquired immune responses *via* regulating cytotoxic T cell development, DC maturation, and NK cell activation (39). HPV-16 VLPs stimulate plasmacytoid DC responses such as releasing cytokines like IL-6, interferon alpha (IFN-α), and tumor necrosis factor alpha (TNF-α) in cervical cancer (40–42). Furthermore, in cervical cancer cases, oncoprotein E7 has been shown to inhibit the expression of genes associated with IFN-α activity, resulting in the inhibition of inflammatory cytokine secretion, dendritic cell maturation, and T lymphocyte-dependent cytotoxic response (43). On the other hand, oncoprotein HPV16 E6 suppresses the expression of MHC-I genes, thereby diminishing MHC-I levels (44). The other HPV oncoprotein, E5, also interferes with the expression site of MHC-I at the cell surface and prevents the recognition of HPV by the immune system (7). According to studies, the production of capsid proteins in the differentiated epithelium is reduced and delayed during HPV infection, resulting in LCs refusing to recognize tumor cells. Moreover, keratinocytes have been shown to act as immunologic cells, such as antigen-presenting cells (APCs), secreting T-helper (Th1) and Th2 cytokines and inducing the cytotoxic response of CD8+ T cells (7). T cells’ immunologic responses are also influenced by antigen binding to the TCR receptor and the creation of the antigen-peptide complex *via* MHC and immune checkpoints that modulate the balance of stimulatory and inhibitory signals. The most crucial immune checkpoints, PD-1/PD-L1 and CTLA-4, have been proven to operate as maintainers of immune response homeostasis (45–47). PD-1 is significantly expressed on the surface of regulatory T lymphocytes rather than on the surface of activated T lymphocytes or tumor-penetrating T lymphocytes (45). Increased PD-L1 expression during malignancy can also allow tumor cells to evade the immune system’s response. Also, CTLA-4 limits the immune system hyperactivity by decreasing T cell function (45). As a result, immune checkpoints could be a therapeutic target for inhibiting the PD-1/PD-L1 and related pathways, which might be a promising strategy for treating cervical cancer (46, 48). As previously stated, the virus evades the immune response through a variety of mechanisms, including increased expression of immune checkpoints, decreased expression of MHC-I, and suppression of IFN activity, which overall leads to the secretion of inflammatory cytokines such as IL-10 and transforming growth factor-beta (TGF-β), creating an ideal environment for immune suppression (49). By phagocytosing damaged cervical cancer cells, LCs also boost the secretion of immunosuppressive cytokines such as TGF-β, IL-10, and IL-13, which suppress DC maturation and stimulate the cytotoxic response of T-cells (7, 50). Overall, targeting immune or cellular pathways that participate in tumor progression can be a suitable therapeutic strategy in malignancies such as cervical cancer.

## 4. Therapeutic approaches in the treatment of HPV-related cancers

Since infection with HPV takes a long time to create precancerous lesions, vaccination does not influence the development of malignancy (51). Besides, preventative vaccines also vary from therapeutic vaccines, which promote the humoral immune response rather than the cellular immune response. These vaccines also boost immune function to prevent and protect against HPV infection but have no therapeutic impact on prior infections or disease (5). Stage IA cervical cancer is recommended to be surgically treated. Surgical and irradiation are used together in stages IB and IIA, while radiation treatment is used alone in stages IIB-IVA. Chemotherapy and local radiotherapy are also utilized in stage IVB (52). Intracavitary brachytherapy and radiation are two other options as well. According to a study, the initial phase of this treatment is to eliminate malignant cervical tissue and the surrounding tumor environment (52). At the same time, other therapies lead to the destruction of secondary deposits in the parametrium, regional lymph nodes, and other small pelvic organs (52). Also, therapeutic vaccinations can trigger the cellular immune response against infected cells, such as precancerous or cervical lesions. In this way, protein and nucleic acid-based vaccines and live vectors are examples of therapeutic vaccines. For instance, ADXS11-011 is one of the most extensively used vaccines for HPV-related cervical cancer, which is a live attenuated *Listeria monocytogenes* (Lm) that causes the release of a fusion protein called HPV-16 E7 (53). ISA101 is another peptide vaccine consisting of 12 long synthetic peptides derived from E6 and E7 HPV-16 (54, 55). Moreover, therapeutic vaccines that target tumor cells with E6 and E7 oncoproteins by stimulating a cellular immune response are examples of immunotherapy, as a result, novel and efficient therapies are required (56). On the other hand, immune checkpoints, such as PD-1, reduce the activity of T cells in the peripheral tissue, inhibit detection of pathogens during the immune response, and also lead to self-tolerance. In addition, the PD-1/PD-L1 cellular pathway can inhibit the T cells’ function in response to tumoral cells and plays an important role in the process of chronic viral infection by preventing the proliferation and function of virus-specific T cells (6). In this way, immunotherapy and immune checkpoint inhibitors have a significant role in a variety of cancers (54). It has been demonstrated that the combination of GX-188E vaccination and pembrolizumab promotes the recruitment of tumor-infiltrating lymphocytes and increases PD-L1 expression in tumor cells (57).

Targeted therapy has recently been proposed as a new treatment strategy. Targeted therapies are divided into two groups: First, small molecules entering the cytoplasm affect cellular pathways such as tyrosine kinases (PI3K/AKT/mTOR pathways), DNA repair mechanisms, and polymerase inhibitors (PARP) (53). Second, monoclonal antibodies bind specifically to ligands and receptors on the cell surface while not penetrating the cells, and then these antibodies destroy cancer cells through antibody-dependent cellular cytotoxicity (ADCC) (58). For example, bevacizumab is used as a monoclonal antibody to inhibit VEGF, and pembrolizumab is used as a PD-1 inhibitor to treat gynecological malignancies (53). Besides, anti-PD-1/PD-L1 monoclonal antibodies as immune checkpoint inhibitors have been shown to be effective in treating incompatibility repair-deficient endometrial cancers, while HPV-related treatments are now being developed for HPV malignancies (53). Currently, it has been suggested that monoclonal antibodies are a new treatment approach for infections caused by hr-HPV E6 and E7 oncoproteins (59).

## 5. Advantages and disadvantages of monoclonal antibody therapies

The development of cancer pharmaceuticals has been supported by increased understanding of cancer genomics, the immunological landscape, and alterations in molecular mechanisms (53). Targeted therapeutics such as monoclonal antibodies and immune checkpoint inhibitors have been developed following these advancements, especially compared to other conventional methods like surgery, chemotherapy, and hormone therapies. However, these therapies have both advantages and disadvantages. In addition to interrupting DNA replication and mitosis, chemotherapy targets the carcinogenic signaling pathway, the immunological microenvironment, and the vascular system in tumor tissue (53). Also, radiation therapy causes the secretion of tumor antigens and the development of a robust immune response in the irradiated area through increased abscopal effect and raising antigen presentation and cytokine secretion (60). Moreover, clinical and preclinical evidence reveals a strong link between intravenous immunoglobulin usage and therapeutic efficacy. These benefits include modulating macrophage polarization toward the M1 phenotype, pro-inflammatory activity, and eventually decreasing tumor cell proliferation (61). However, treatment with monoclonal antibodies may lead to several disorders. For example, following immunosuppressants that do not target tumor-specific T cells, immune response activation against self-antigens leads to a non-tumor-specific immune response. Subsequently, following immunotherapy, immune-related side effects in healthy tissue often result in skin disorders such as pruritus and mucositis (62). For instance, in patients treated with the anti-CTLA-4 drug, 68% experienced skin disorders; whereas in patients treated with ipilimumab, 40% reported gastrointestinal disturbances such as colitis and diarrhea (62). Other side effects, including hepatotoxicity, endocrinopathy, and pneumonitis, are less commonly reported. In addition, renal toxicity, neurotoxicity, cardiovascular toxicity, pancreatitis, blood disorders, and ocular manifestations are less common compared to the mentioned side effects (63).

Furthermore, gastrointestinal, endocrine, and pulmonary disorders are the side effects of balstilimab (64), and zalifrelimab is associated with hypothyroidism and hyperthyroidism (65). Long-term use of durvalumab has also been reported to cause side effects such as pneumonitis, hepatitis, colitis or diarrhea, hypothyroidism, adrenal insufficiency, hypophysitis or hypopituitarism, type 1 diabetes, nephritis, and skin rash or dermatitis. Other side effects such as diarrhea, fatigue, and hypokalemia were observed in 35%, 25%, and 25% of cases, respectively (13).

## 6. The role of monoclonal antibodies in cancer diagnosis

It was demonstrated that antibodies can be useful for the treatment and diagnosis of tumors after the antibodies discovery and their function (66). Antibodies can also be employed to directly target particular tumor-associated antigens (TAA) because of their specialized function (67).

More than 100 distinct monoclonal antibody products are currently available, and they are mostly employed for therapeutic and standard diagnostic procedures (68). Furthermore, monoclonal antibodies are crucial for identifying proteins, carbohydrates, and nucleic acids in the field of biomedical research. As a result of their usage, molecules involved in cell proliferation and differentiation have been discovered (68). These antibodies have been recognized as crucial tools for biomedical research, microbiology, the detection of herpes simplex, hepatitis, AIDS, and other diseases, and finally the diagnosis and treatment of cancer and other illnesses (68).

Tumor-associated antigens, which are different targets of therapeutic antibodies, can be categorized based on the type of cancer they target (67). In this regard, malignancies with cancerous tumors are targeted through numerous antigens that are categorized into distinct groups based on their function, whereas hematological cancers are targeted through CD markers such as CD20, CD30, CD33, and CD52 (66). For instance, the EGFR as a TAA can be a therapeutic target for cancer therapy. In other words, EGFR-specific antibodies result in disruption of the receptor’s function, suppression of tumor development, and ultimately, Fc signaling by innate immune cells to destroy tumor cells (67). According to reports, target antigens may also be present in tissues including blood vessels, stroma, and extracellular matrix in addition to the tumor environment (66, 69). Tumor-associated antigens, which are different targets of therapeutic antibodies, can be categorized based on the type of cancer they target (70). In this approach, the capacity of tumor cells to create vascular systems is limited after blocking the receptor or trapping the appropriate ligand, and as a result, the ability to grow and spread is diminished (70). In addition, it is possible to target the immune system when treating cancer. As a cell surface receptor on T cells, CTLA-4 leads to regulation of T cells function (67, 71). One such example is the monoclonal antibody ipilimumab, which prevents cytotoxic T cells from producing CTLA-4, hence suppressing CTLA-4 and increasing T cell anticancer activity (71).

## 7. Functions of monoclonal antibodies against cervical cancer

The application of immune checkpoint inhibitors is an effective technique for preventing cancer progression by targeting the immune system. In this regard, targeting CTLA-4 and PD-1 on the activated T cells’ surface leads to the immunologic tolerance of cancer cells in the tumor setting (6). On the other hand, it has been illustrated that the overexpression of PD-1 and PD-L1 on the cervical DCs and T lymphocytes is correlated to greater cytological abnormalities and high-risk HPV positivity in individuals with CIN as well as a poor prognosis in cervical cancer patients (72). Monoclonal antibodies including: anti-CTLA 4, anti-PD-1, anti-PDL-1, anti-EGFR, and anti-VEGF that are currently available for targeted therapy. In the following, we have reviewed their importance and targeted pathways (7).

### 7.1 Anti-PD-1/PD-L1,2

The overexpression of PD-1 (CD279), a molecule on the surface of activated T lymphocytes, especially T-regs, is involved in immune homeostasis. On the other hand, its ligands PD-L1 and PD-L2, expressed on the surface of tumor cells and APCs, have been associated with cancer progression (73). The interaction between PD-1 and PD-L1 suppresses T lymphocytes’ proliferation, enhances CTL degradation, cellular signaling pathway impairment, and apoptosis (73). In other words, increased PD-1 expression on the T cells’ surface during persistent and chronic infection has the reverse effect on infection control. Upon malignancy, the overexpression of PD-L1 on the surface of tumor cells reduces the cytotoxic function of CD8+ T lymphocytes in the rhizomatous environment, thereby inhibiting apoptosis (74). Besides, PD-L1 can be expressed primarily on the surface of tumor cells, either locally by IFNs or by stromal cells such as macrophages (73). In this context, PD-1-specific monoclonal antibodies like pembrolizumab, nivolumab, and cemiplimab, and PD-L1-specific antibodies like atezolizumab, dorvalumab, and avelumab, have advantages against tumors and malignancies (75).

#### 7.1.1 Pembrolizumab

Pembrolizumab is a highly specialized human monoclonal antibody with the molecular formula C6504H10004N1716O2036S46 that is more therapeutically efficacious than human IgG4 caused by changes in the Ser228Pro sequence in the Fc region (76). Due to the Ser to Pro change at position 228 and the high molecular weight of this antibody (148.9 kDa), compared to the most abundant light and heavy chains (50.7 kDa and 23.7 kDa), the immunotherapy’s instability and toxicity are minimized (6). Pembrolizumab also has two steady and variable regions; the variable area binds to PD-1 with great affinity and specificity, preventing PD-1/PD-L1/L2 interactions (77). Pembrolizumab’s constant region also causes the activation of the cellular immune system and complement due to its low affinity for C1q and Fc receptors (78). The PD-1 and pembrolizumab interaction is correlated to the interaction of pembrolizumab with PD-1 flexible loops, which do not participate in the PD-L1 exchange (6). Following pembrolizumab binding to the C and C’ strands in PD-1, the PD-L1 junction is occupied and minor structural alterations in PD-1 occur, so it has been shown that pembrolizumab’s effect on other PD-1 ligands is different (78, 79).

Due to intravenous injection, pembrolizumab has not been reported to interact with plasma proteins. In addition, it is catabolized into small peptides and single amino acids through general protein degradation pathways and has a half-life of 26 days. Factors such as age, gender, mild to moderate renal insufficiency, liver failure, and cancer do not interfere with the clearance of pembrolizumab (0.23 liters per day). Given the lack of dependence of pembrolizumab metabolism on the kidneys and liver, there have been reported to be no immune disorders in cases of moderate or severe renal and hepatic insufficiency (6). On the other hand, pembrolizumab’s effectiveness causes the recovery of tumor-specific T cytotoxic cells in the tumor microenvironment and reactivates the anti-tumor immune system. This monoclonal antibody has been shown to have anti-cancer effects in advanced melanoma, non-small cell lung cancer, Hodgkin’s lymphoma, urothelial carcinoma, and squamous cell carcinoma of the head and neck, as well as a 14.3% immune response during treatment of recurrent or metastatic cervical malignancy. Following the KEYNOTE-158 study, the Food and Drug Administration (FDA) approved pembrolizumab for the treatment of advanced cervical cancer on June 12, 2018, and it is recommended as a second-line treatment for PD-L1 positive cervical cancers that have progressed following chemoresistance, as well as in 2021 it has been approved as the first-line treatment for cervical malignancies (57).

#### 7.1.2 Cemiplimab

Cemiplimab is a recombinant human IgG4 monoclonal antibody that binds to the PD-1 receptor and blocks the PD-L1 and PD-L2 ligands. This antibody inhibits the immune response and shrinks the tumor cells (80). In this regard, cemiplimab was approved in 2018 in the United States for the treatment of patients with metastatic or advanced SCC who did not respond to radiation therapy or surgery. In a phase II clinical trial (NCT02760498) studying cemiplimab (81), the patients showed a 47% immune response after 1.9 months (80). It is noteworthy that cemiplimab entered directly into the phase III clinical trials following the results obtained in phase I and II studies (82). Moreover, clinical results suggest that, in patients with recurrent or metastatic cervical cancer, cemiplimab treatment leads to an appropriate immune response, where 66% of cervical cancer cases have been recovered following phase I clinical trials of cemiplimab in human’s study (83). Furthermore, cemiplimab has the same safety and clinical benefits as other PD-1 inhibitors in patients with metastatic or recurrent cervical cancer who are resistant to the combination chemotherapy (platinum and taxane). It is also approved as the first-line treatment after platinum-based chemotherapy with significant survival effects (NCT03257267) (84). Besides, the clinical activity of cemiplimab in phase I clinical trials is directly related to the histology of cervical cancer.

#### 7.1.3 Durvalumab

Durvalumab (ImfinziTM; AstraZeneca) is a human monoclonal antibody that suppresses PD-L1, prevents it from interacting with PD-1, and increases the cytotoxic and anti-tumor activity of T cells (13). This antibody was approved by the FDA on May 1, 2017, due to its ORR in treating local metastatic or advanced urothelial cancer (85). In addition, the impact of durvalumab on combinational therapies such as radiotherapy, ADXS11-001, and tremolimumab was evaluated in clinical terials in cervical cancer cases (86).

#### 7.1.4 Atezolizumab

Atezolizumab, as an IgG1 subclass human monoclonal antibody, leads to increased activity of cytotoxic T cells and tumor-infiltrating lymphocytes and also interacts with the PD-L1 ligand (13). On the other hand, this antibody inhibits PD-L1 and PD-1 interaction, which disrupts the activity of the cellular immune system and T lymphocytes (87). Besides, atezolizumab is the first FDA-approved anti-PD-L1 drug currently prescribed for urothelial carcinomas and non-small cell lung cancer (NSCLC) (86, 88, 89). This group of antibodies restores the anti-tumor immune response by impairing the immune response induced by PD-L1/PD-1 without causing antibody-dependent cytotoxicity (13, 45). In this way, in a study conducted by Petrylak et al., on patients with metastatic urothelial malignancy, atezolizumab resulted in the treatment and recovery of pre-treated patients; however, its long-term clinical features are unknown (90).

Furthermore, atezolizumab has been studied in combination therapies such as chemotherapy, radiation therapy, and bevacizumab (NCT03614949, NCT03340376, NCT02921269, NCT03073525) (86).

#### 7.1.5 Avelumab

The FDA approved avelumab on March 23, 2017, for Merkel cell carcinoma treatment (91). It is a completely human monoclonal antibody of the IgG1-λ class that blocks PD-L1 and inhibits its interaction with PD-1. Besides, avelumab was approved on May 9, 2017, for the treatment of metastatic or advanced topical urothelial malignancy (92). In this regard, some clinical trials (NCT03260023 and NCT03217747) were performed to evaluate the effect of avelumab on cervical cancer (86). Le Tourneau et al., showed that the co-treatment with avelumab and TG4001 induced anti-tumor activity in cervical cancer patients. Moreover, in patients with poor baseline immunity, changes in the immune response were recorded (93).

#### 7.1.6 Nivolumab

Another human IgG4-κ subclass monoclonal antibody that inhibits the PD-1 immune checkpoint is nivolumab (86). This antibody stimulates immune responses such as T cell function, anti-tumor response, and apoptosis by binding to the PD-1 receptor while preventing it from interacting with its specific ligands (PD-L1 and PD-L2) (94). Nivolumab has also been demonstrated to be less toxic and has significantly better clinical activity in cervical cancer. In addition, this monoclonal antibody has been examined in phase II clinical trials in advanced or recurring cervical cancer patients. Encouragement evidence showed that following treatment with nivolumab, 20% of patients had a significant immune response (95). Nivolumab therapy has been approved for disorders such as metastatic melanoma, SCC, renal cell carcinoma, and cervical cancer (94). However, the extent of nivolumab’s activity and the HPV infection progression have yielded disparate outcomes. Furthermore, compared to monotherapy, the combination of two targeted therapies or immunotherapy has recently been demonstrated to be significantly effective in the suppression of malignancies such as cervical cancer (95).

#### 7.1.7 Balstilimab

Another monoclonal antibody that targets PD-1 and its interaction with PD-L1 is balstilimab (AGEN2034), which is a human IgG4 monoclonal antibody (96). Following the use of balstilimab, the T cell receptor signaling pathway increases when T cells respond to tumor-associated antigens. Also, in phase I clinical trials of balstilimab, it was observed that the rates of toxicity associated with treatment are 3.4% and 11.8%, whereas the rates of reactions related to infusion, gastrointestinal disorders, and endocrinology are lower than other therapies (64). Besides, phase II clinical trials (NCT03495882) were performed and evidence showed that the combination of balstilimab and zalifrelimab evaluated the effect of immune checkpoint inhibitors in cervical cancer individuals with more appropriate clinical outcomes (65). Also, this combination was conducted to promote efficacy and anti-tumor activity in patients with advanced, recurrent, and metastatic cervical cancer. In addition to the above, in a phase II study on evaluating the effectiveness of balstilimab alone (NCT03104699), it was suggested that in patients with recurrent and metastatic cervical malignancy, treatment with balstilimab led to increased activity and optimal immune tolerance compared to platinum-based chemotherapy (65).

### 7.2 Anti-CTLA-4

T lymphocyte activity and proliferation diminish after CD28 and CTLA-4 binding, resulting in a stable state. T cells reactivated by reducing CD28 and CTLA-4 binding negatively affect T-reg cell activity because CTLA-4 is highly abundant in tumor cells (73, 97). Anti-CTLA-4 antibodies have been observed to reactivate as T lymphocyte agonists, regardless of the tumor T cells’ specificity (73).

#### 7.2.1 Ipilimumab

In humans, ipilimumab is an IgG1-κ monoclonal antibody that targets CTLA-4 (98). Bristol-Myers Squibb launched ipilimumab under the Yervoy brand and was approved by the FDA in 2011, initiating a new era in immunotherapy (62).

Ipilimumab was investigated as the first immune checkpoint inhibitor and the first antagonist for melanoma before pembrolizumab. Combination therapy with pembrolizumab was discovered to have a significant effect on the neoplastic process (71, 99). Ipilimumab has been shown to stimulate the immune response in peripheral blood; however, patients with cancer were not significantly affected due to the lack of activated immune cell induction and access to tumor cells (100).

Furthermore, factors such as hypoxia and prior pelvic radiotherapy in immunocompromised cells in cervical cancer patients were reduced after ipilimumab treatment (100, 101). On the other hand, monotherapy with ipilimumab and CTLA-4 inhibition was not approved in cervical cancer patients due to a lack of targeted treatment. Also, given that various experiments and expression of some markers on circulating T lymphocytes’ surface and the expression of PD-1 inhibitor molecules on T cells’ surfaces following ipilimumab administration, led to a new perspective for target therapy with PD-1 monoclonal antibody inhibitors in cervical cancer patients (7). Nevertheless, other studies are being performed on this monoclonal antibody’s utilization in cervical cancer cases.

#### 7.2.2 Tremelimumab

Another monoclonal antibody against CTLA-4 is tremelimumab, which is an IgG2 human monoclonal antibody developed by AstraZeneca (62). Although tremelimumab monotherapy has not been shown to improve patients in any clinical trials, it is considered effective in combination with other inhibitors or anti-neoplastic drugs (62).

#### 7.2.3 Zalifrelimab

Zalifrelimab is another type of human IgG1 monoclonal antibody that targets CTLA-4. In PD-1 resistant solid tumors, zaliferlimab as a single therapy leads to enhanced activity and appropriate tolerance. It is worth noting that zalifrelimab has been considered effective in combination with immunotherapy (102). It has been reported that zalifrelimab potentiates other immunomodulatory antibodies’ functions, such as balstilimab, both *in vitro* and *in vivo*, and also, in combination with PD-1 inhibitors, increases the activity and proliferation of T cells in non-human mammals (103).

### 7.3 Anti-VEGF

Given that HPV-16 E5 oncoprotein regulates vascular endothelial growth factor (VEGF) overexpression *via* the EGFR-MEK1/2, and PI3K-AKT pathways, it plays a crucial role in tumor progression (104). Because the EGFR gene is overexpressed in 80% of cervical cancer tumors, an anti-viral targeting E6/E7/E5 oncoproteins or related signaling pathways, such as an anti-EGFR agent, can play a critical role in preventing cancer progression (105, 106).

#### 7.3.1 Bevacizumab

Bevacizumab or Avastin is a human monoclonal antibody that inhibits VEGF and angiogenesis in tumor cells (107). Higher levels of hypoxia-induced factor- and VEGF production have been linked to increased angiogenesis in cancer, making angiogenesis a promising target in advanced cervical cancer (108).

Furthermore, bevacizumab has been recommended in patients with persistent, recurring, and metastatic cervical cancer in combination with chemotherapy as a first-line treatment, resulting in enhanced survival and recovery (109, 110). Based on the results of the GOG-240 phase III study of bevacizumab in 2014, the combinationtherapy of bevacizumab with chemotherapy drugs was found to improve median overall survival by an average of 3.7 months in patients with recurrent, persistent, or metastatic cervical cancer (111). In addition, when bevacizumab was combined with chemotherapy, the therapeutic effects were more robust than single chemotherapy (107). Moreover, TNP-470, an angiogenesis inhibitor produced by *Aspergillus fumigatus* Fresnius, was demonstrated to be therapeutic in phase I clinical trials (112), and bevacizumab was shown to be therapeutic in phase II clinical trials (113). Overall, inhibiting VEGF in the course of cervical cancer is more successful than inhibiting VEGF in ovarian cancer; in other words, bevacizumab has a higher inhibitory effect on cervical cancer than ovarian cancer (109).

#### 7.3.2 Cetuximab

Cetuximab (Cx) (IMC-C225; Erbitux) is an IgG1 human monoclonal antibody that functions as an anti-EGFR ligand. This monoclonal antibody has a cytotoxic impact when combined with chemotherapeutic medications, inhibiting angiogenesis and VEGF expression (114). It has been suggested that Cx combined with cidofovir (Cd), leads to inhibition of E6/E7 oncoproteins and impairs the p53/EGR1/EGFR/VEGF pathways (106). Moreover, cetuximab promotes the cytoplasmic suppression of DNA-dependent protein kinase, which supports the repair of non-homologous terminal DNA binding pathways (115). It is worth noting that, a higher expression level of EGR-1 in tumor tissue can control human telomerase reverse transcriptase enzyme activity and p53 function alteration (116, 117). In this regard, Myllynen et al., showed that EGFR leads to double-strand break (DSB) DNA regeneration *via* both non-homologous end joining (NHEJ) and homologous recombination (118). The suppression of the EGFR signaling pathway causes the inhibition of DSB in such cases. Moreover, inhibition of EGR-1 expression decreases the proliferation and migration of microvascular endothelial cells and reduces VEGF expression and tumor angiogenesis (118). On the other hand, cetuximab monotherapy leads to radiation therapy sensitization in cervical cancer cases.

### 7.4 Anti-TIGIT

T cell immunoreceptor with Ig and ITIM domains (TIGIT), as a type of immune-checkpoint inhibitor receptor, is involved in the control of tumor immune surveillance (119). In addition, TIGIT is expressed on the surface of T cells and NK cells and plays a significant role in their activity and maturation (119). Also, this immune-checkpoint inhibitor competes with immunoactivator receptor CD226 (DNAM-1) to bind to ligands such as CD155 (PVR or polio receptor) and CD112 (nectin-2 or PVRL2) (119). In other words, by interacting with three ligands (CD155, CD112, and CD113) expressed on the surface of tumor cells, TIGIT causes the immune system to be suppressed (120). In this regard, Liu et al., in 2022, demonstrated that TIGIT is a novel therapeutic target in cervical malignancy; additionally, it plays a significant role in preclinical models of cervical cancer following inhibition of TIGIT by monoclonal antibodies, both alone and in combination with anti-PD-1/PD-L1 antibodies (121). On the other hand, similar to PD-1/PD-L1, TIGIT plays a role in inhibiting the tumor immune system and both are upregulated in different types of malignancies (119). It is worth mentioning that in preclinical models, anti-TIGIT antibodies are synergistic with anti-PD-1/PD-L1 antibodies (122–124). For example, etigilimab (OMP-313R12), as a type of anti-TIGIT antibod, leads to inhibition of the growth of a mouse colon carcinoma model (CT26 WT) (125). It was also shown that this antibody in combination therapy with anti-PD-L1 in mouse models led to increased recovery of malignancy compared to the control group (124). In this regard, clinical trials related to anti-TIGIT as a combonation or monotherapy are ongoing (126).

#### 7.4.1 Tiragolumab

Recently, it has been reported that patients treated with the human IgG1 monoclonal antibody tiragolumab, following a combination treatment strategy, showed safe and effective results, which is now in the final stages of clinical development (127). Also, in today’s invetigations, all anti-TIGIT monoclonal antibodies have a key common feature such as high binding affinity to the receptor and target molecule. In other words, these monoclonal antibodies lead to the activation of the T cell and NK cell signaling pathways in malignancies by inhibiting the binding of TIGIT to CD155. Also, tiragolumab leads to the preservation of antibody-coated tumor cells through the antibody-dependent cytotoxicity (ADCC) pathway. On the other hand, the TIGIT-PVR pathway is one of the targets of other monoclonal antibodies. In this regard, the antibody that inhibits the TIGIT-PVR pathway is being commercially developed in 2020 (126). Also, the FDA in 2021 approved the combined treatment of tiragulumab with atezolizumab as the first-line treatment for patients with non-small cell lung cancer (NSCLC) malignancy and tumor cells with high expression of PD-L1 without mutations in the tumor gene EGFR (126). Tiragolumab is in development stages with two clinical trials in phase III and two clinical trials in phase II (126).

## 7. Clinical use of monoclonal antibodies against cervical cancer

As discussed in the previous sections, most recent clinical trials have been predicated on the use of monoclonal antibodies and their combinations to inhibit PD-L1/PD-1 interaction and prevent of T lymphocyte death. Also, in 20% of metastatic epithelial malignancy cases, following the use of these monoclonal antibodies for one to two years resulted in an immunological response (128). In this way, in a preclinical trial in mice with malignancies, it was discovered that the tumor size and progression were dramatically reduced after the combination of anti-CTLA-4 monoclonal antibodies with HPV E6/E7-specific vaccinations. Moreover, following the combinationtherapy, 41BB activation induces the activation of cytotoxic T cells. In the co-administration of the anti-41BB agonist with the HPV E7 DNA vaccine and recombinant IL-2, survival was enhanced compared to the HPV E7 DNA vaccine alone in clinical research (95). In other words, 41BB is a receptor implicated in the CTLA-4 pathway, which leads to the activation of T cells and APCs, as well as the secretion of pro-inflammatory cytokines such as IL-12 and IL-6 (129).

Furthermore, in individuals with advanced cervical cancer, a 33% recovery rate was reported over 10.3 months following co-treatment with vaccines and immune checkpoint inhibitors. For instance, following the combination of the GX-188E DNA vaccine with pembrolizumab (Merck, NCT03444376), there was a significant improvement over monotherapy (95). In this way, Won Youn et al., showed that patients with recurring or advanced cervical cancer had safe treatment with the GX-188E therapeutic vaccination combined with pembrolizumab, and any side effects were controlled. This intermediate analysis revealed preliminary anti-tumor activity in the combination therapy, suggesting a new possible therapeutic approach for this patient group. This trial is ongoing (57).

On this basis, the use of monoclonal antibodies in combination with therapeutic vaccines can increase the production of pro-inflammatory cytokines, decrease the level of regulatory T cells, and macrophages, and inhibit antigen-specific T lymphocytes (95). For example, in combination therapy of GX-188E vaccine with pembrolizumab, showed an acceptable anti-tumor response in patients with recurrent, advanced, or metastatic cervical cancer (57). Remarkably, this treatment in patients with advanced cervical cancer, even with two courses of chemotherapy, leads to the proper induction of E6 and E7 HPV-specific T lymphocytes (57). Furthermore, another study on cervical cancer patients revealed that the tolerable response rate for anti-PD-1/PD-L1 monotherapy varied from 8.8% to 26.3%, with the highest rate associated with tumor cells with PD-L1 overexpression (100, 130, 131). On this premise, pembrolizumab was approved by FDA with 14.6% effectiveness on tumor cells expressing PD-L1 in the KEYNOTE-158 trial (NCT02628067). In the study conducted by O’Malley et al., in 2022, it has been shown that pembrolizumab improved health-related quality of life (HRQoL) in subjects with advanced MSI-H/dMMR endometrial malignancy, which is in line with the results of the KEYNOTE-158 study and the use of pembrolizumab (132). In addition, Shapira-Frommer et al. showed that in patients with SCC of the vulvar, following monotherapy with pembrolizumab and regardless of PD-L1 expression level in tumor cells, a good response was created. Overall, they reported that, similar to the KEYNOTE-158 results, pembrolizumab works well (133). On the other hand, pembrolizumab can remove tumor cells due to the reinvigoration of exhausted T cells. In this regard, following a phase II study of patients with recurrent or advanced cervical malignancies that significantly expressed PD-L1, 14% of patients were sensitive to chemotherapy and responded appropriately to treatment (134). Also, KEYNOTE-028, conducted by Frenel et al., in 2017, in addition to KEYNOTE-158, utilized pembrolizumab in a phase I clinical trial with an ORR of 17% in recurrent and metastatic cervical cancer. In terms of toxicity and side effects, 20.8% of patients experienced grade 3 adverse events (AEs) (NCICTCAE 3.0) but no grade 4 AEs (75). Pembrolizumab clinical trial studies, on the other hand, investigated the impact of six INF-γ genes on patient tumor prototypes, including Indoleamine 2, 3-dioxygenase 1 (IDO1), C-X-C motif chemokine (CXCL)10, CXCL9, Human Leukocyte Antigen (HLA)-DRA, Signal transducer and activator of transcription (STAT) 1, and IFN-γ (135–137). These findings suggest that the immune response to PD-1 inhibitors may be related to the presence of an acquired immune response following INF-γ production in a variety of cancers, including advanced melanoma, head and neck squamous cell carcinoma (HNSCC), anal canal, biliary, gastrointestinal, colon, esophageal, and ovarian cancers (54).

In another clinical trial, the cure rate of HPV malignancies with ISA101 and nivolumab co-treatment was reported to be 33% (138).

Furthermore, Massarelli et al., in a study, found that in cases with advanced and metastatic HPV-induced malignancy, the combination of nivolumab with synthetic long-lived HPV peptides (IAS101) along with Montanide (Seppic) had an acceptable response (NCT02426892) (138). Alos, Sousa LG, in 2022, illustrated that (In long-term follow-up, the efficacy of ISA101 with nivolumab is still encouraging. Response was predicted by increased PD-1+ T cell and macrophage infiltration. Immune infiltration and interferon response gene set enrichment significantly predicted therapeutic response. This approach is currently being evaluated in a randomized experiment, which is also investigating the correlations between the immune response to the combination of nivolumab and ISA101 against nivolumab monotherapy.) (139).

In other clinical trials (NCT02921269) conducted in phase II, in patients with recurrent cervical malignancy, atezolizumab was used as a second-line treatment (13). The addition of bevacizumab to PD-L1 blocking did not appear to improve the ORR in cervical cancer, hence the bevacizumab and atezolizumab combination did not achieve the predefined effectiveness target (111).

Also, another study in phase III on patients with recurrent, stable, or metastatic infections (NCT03556839) reported enhanced recovery following the combination of chemotherapy drugs such as cisplatin or paclitaxel (a chemotherapy drug) plus monoclonal antibodies such as atezolizumab or bevacizumab (13). It has been reported that the combination of chemotherapy along with monoclonal antibodies such as bevacizumab and atezolizumab leads to an increase in the survival of patients with metastatic, recurrent or persistent cervical cancer and ultimately a new approach for treatment (140). In a phase II clinical study (NCT02921269) conducted by Friedman et al., following the combination of bevacizumab with atezolizumab in patients with advanced cervical cancer, the results suggested that the safety profiles associated with both drugs were consistent (111). However, no increase in overall response rates (ORR) was observed following the combination of these two monoclonal antibodies. The combined regimen of vaccines in phase I/II, carboplatin, paclitaxel, and with or without bevacizumab in cervical cancer is being evaluated to find a selective therapeutic approach for CCs (NCT02128126) (53).

In another phase III clinical trial, durvalumab plus cisplatin was evaluated for two years in advanced cervical cancer patients (13). Also noted is that in the phase I study in the metastatic stage of cervical cancer, the combination of durvalumab and tremelimumab (NCT01975831) demonstrated an acceptable anti-neoplastic effect (141). In a phase I-II research published in 2017, Hollebecque et al. (CheckMate 358) nivolumab was used to treat recurrent and metastatic cervical cancer. According to their findings, the ORR was 26.3%, while the disease control rate was 70.8%. Hyponatremia, syncope, diarrhea, and liver damage were also reported as grade 3 and 4 side effects (75). According to research by Ferris et al. in 2021, neoadjuvant nivolumab causes pathological regression in both HPV positive (23.5%) and HPV negative (5.9%) cancers. Nivolumab neoadjuvant is safe, and to maximize efficacy, combination neoadjuvant therapy regimens and continuous treatment after surgery for malignant tumors were advised (142).

Furthermore, it has been illustrated that combining two monoclonal antibodies, ipilimumab and nivolumab, has a more significant impact than ipilimumab monotherapy. However, to safely integrate these two monoclonal antibodies, the dose of ipilimumab has to be reduced (141).

## 8. Future perspective on monoclonal antibodies suggested in cervical cancer

As mentioned in the previous section, HPV, through integration into the host genome and disruption of the host transcription process through the HPV E2 protein, leads to increased overexpression of HPV E6 and E7 and increased malignancy (143, 144). As a result, the knockdown of HPV E6 and E7 expression or activation can reduce malignancy and thus become a target for specific treatment of cervical cancer (59). Alongside radioimmunotherapy (RIT), mAbs are being developed as a new approach to combat E6 and E7 oncoproteins (145, 146). The results of studies by Jiang et al., for the monoclonal antibodies design associated with HPV E6 and E7 showed that these antibodies, in turn, inhibit the growth of tumor cells specifically showed that these antibodies have less toxicity and more impact and specificity (59). Also, L1 and L2 capsid proteins can be ideal targets for treatment because they are located on the virus capsid’s surface and play a major role in virus and host cell interaction. L1 proteins have distinct differences in various HPV types, which means that antibodies produced against one type of HPV infection that is included in vaccines do not protect against other HPV infection types (26, 27). Also, in natural infections in people with activated adequate immune systems, antibodies are produced against the L1 capsid protein, which this type of antibody can neutralize the virus. Monoclonal antibodies against L1 protein can prevent HPV infection in various *in vitro* and *in vivo* models (24, 25). Regarding this, in a study conducted by Martha et al., monoclonal antibodies against HPV L1 VLP in the carcinogenic variants HPV31, HPV33, HPV45, HPV52, and HPV58 showed several characteristics, including specificity, non-interference with each other if combined, sufficient affinity for antigen binding, and a long half-life (147). However, long-term treatment may cause cystitis, proctitis, ovarian failure, and chronic pelvic pain (107). Given that cervical cancer is considered a therapeutic candidate in immunotherapy, it has been predicted that anti-killer cell immunoglobulin-like receptor (KIR) antibodies are a new treatment method in treating cervical cancer (7). Nevertheless, there is no specific treatment outside of clinical trials due to the various advances in the mechanism of HPV infection and the progression of cervical cancer related to HPV infection. In this way, more research is needed (59).

## 9. Discussion

Presently, targeting the immune system is a suitable strategy for treating cervical cancer, in which immune checkpoint inhibitors play a crucial role. For example, activated T cells expressing CTLA-4 and PD-1 develop immunological tolerance to cancer cells in a resorbable area. In other words, targeting immune checkpoints leads to treating malignancies such as cervical cancer. Recently, immunotherapy agents have been suggested for cervical carcinomas, such as anti-CTLA-4, anti-PD-1, anti-PD-L1, anti-VEGF, and anti-EGFR agents. In this regard, the use of single monoclonal antibodies against CTLA-4 does not elicit a favorable response compared to the combination of anti-CTLA-4 and PD-1 antibodies. In other words, treatment with anti-CTLA-4 leads to the expression of PD-1 on the tumor cell surface. In addition, the expression of PD-1 and PD-L1, in turn, is related to the response to immunotherapy (7). Also, cytological changes and CIN patients with hr-HPV positivity are associated with increased expression of PD-1 and PD-L1 on cervical T cells and dendritic cells, respectively. As a result, the reliability of this treatment depends on the expression of PD-1 and PD-L1 on T cells and tumor cells. Since CTLA-4 inhibits T cell activity in secondary lymphatic organs and PD-1/PD-L1 regulates T cell function in malignant tissues and the tumor microenvironment, PD-1/PD-L1 inhibitors due to the specificity of the PD-1/PD-L1 signaling pathway lead to minimal damage to healthy tissue. Also, despite the different clinical efficacy in cervical cancer carcinoma, inhibition of PD-1/PD-L1 or CTLA-4 signaling pathways is being studied alone or in combination with strategies such as chemotherapy and localized into the tumor.

## Authors contributions

HB: Conceived the idea for this manuscript, Project administration, Edited subsequent drafts. PA: Literature search, Design of the figure and tables, Manuscript preparation, Edite Manuscript. NH: Conceived the idea for this manuscript, Review of the manuscript. HF: Edit and review the manuscript. All authors contributed to the article and approved the submitted version.

## Acknowledgment

This project was supported by the Immunology Research Center, Tabriz University of Medical Sciences, Tabriz, Iran.

## Conflict of interest

The authors declare that the research was conducted in the absence of any commercial or financial relationships that could be construed as a potential conflict of interest.

## Publisher’s note

All claims expressed in this article are solely those of the authors and do not necessarily represent those of their affiliated organizations, or those of the publisher, the editors and the reviewers. Any product that may be evaluated in this article, or claim that may be made by its manufacturer, is not guaranteed or endorsed by the publisher.

## Abbreviations

APCs, Antigen-presenting cells; ADCC, Antibody-dependent cellular cytotoxicity; CTLA-4, Cytotoxic T-lymphocyte-associated protein-4; CIN, Cervical intraepithelial neoplasia; Cd, Cidofovir; CXCL, C-X-C motif chemokine; DC, Dendritic cells; DSB, Double-strand breaks; ERK, Extracellular signal-regulated kinase; EGFR, Epidermal growth factor receptor; FDA, Food and Drug Administration; HPV, Human papillomavirus; HLA, Human Leukocyte Antigen; HNSCC, Head and neck squamous cell carcinoma; IFN-α, Interferon alpha; IDO1, Indoleamine 2, 3-dioxygenase 1; KIR, Killer cells immunoglobulin-like receptor; LC, Langerhans cells; MHC, Major histocompatibility complex; MAPK, Mitogen-activated protein kinase; NK, Natural killer cells; NKT, Natural killer T cells; NSCLC, Non-small cell lung cancer; NHEJ, Non-homologous end joining; PD-L1, Programmed death-ligand-1; PI3K/Akt, Phosphatidylinositol-3-Kinase/Protein kinase B; pRb, Retinoblastoma protein; RIT, Radioimmunotherapy; SCC, Squamous cell carcinoma; STAT, Signal transducer and activator of transcription; TNF-α, Tumor necrosis factor alpha; Th1, T-helper; TGF-β, Transforming growth factor-beta; VEGF, Vascular endothelial growth factor; VLP, Virus-like particles; WHO, World Health Organization; Wnt/β-catenin, Wingless/int.
