# Supplementary material for: Monoclonal antibodies in cervical malignancy-related HPV
Source: Front Oncol. 2022 Oct 6;12:904790. doi: 10.3389/fonc.2022.904790 (PMC9582116; doi:10.3389/fonc.2022.904790)
Supplement: Supplementary file 1 [file Table_1.docx]

**Supplementary data**

**Table 1.** Clinical trials related to the effect of monoclonal antibodies on different stages of cervical cancer, all of which are in the recruiting and not recruiting status.

| **Drug/Intervention** | **Aim of study** | **Target** | **Stage** | **Type of tumor** | **Phase** | **Identifier ID** |
| --- | --- | --- | --- | --- | --- | --- |
| Sintilimab combined with concurrent chemoradiation therapy | Determine the efficacy of sintilimab combined with concurrent chemoradiation therapy | PD-1 | IIA2-IVA | Locally  advanced cervical cancer | II | NCT05105672 |
| Balstilimab Zalifrelimab | Evaluation of efficacy and pharmacokinetics of Balstilimab as monotherapy or combination therapy with Zalifrelimab | PD-1  and  CTLA-4 | - | Recurrent or advanced cervical cancer after first line platinum-based chemotherapy | II | NCT05033132 |
| Pembrolizumab/  Vibostolimab Co-Formulation with or without other anticancer therapies | Evaluation of efficacy and tolerability of the combination of pembrolizumab and vibustolimab (MK-7684A) with or without other anticancer drugs in individuals with solid tumors | PD-LI | - | Solid tumors such as locally recurrent unresectable or metastatic cervical cancer | II | NCT05007106 |
| Camrelizumab with Apatinib versus chemotherapy with Bevacizumab | Comparison of the first-line treatment effectiveness with camrelizumab plus apatinib with the efficacy of paclitaxel and cisplatin / carboplatin plus bevacizumab | PD-1  VEGFR2 | - | IVB cervical cancer,  recurrent and persistent cervical cancer | II | NCT04974944 |
| Camrelizumab plus  Cisplatin or Carboplatin | Evaluation of the efficacy of camerlizumab with chemotherapy and radiotherapy | PD-1 | - | Cervical cancer patient who developed abdominal aortic lymph node metastasis after pelvic wall recurrence surgery. | II | NCT04974827 |
| Sintilimab | Evaluation of the effectiveness of neoadjuvant chemotherapy with CCRT following adjuvant chemotherapy and anti-PD-1 antibody | PD-1 | IIIC2-IVB | Cervical cancer | II | NCT04918628 |
| Camrelizumab with chemoradiotherapy | Evaluation of the effectiveness of camelizumab with chemotherapy radiotherapy | - | - | Recurrent or metastatic cervical cancer (locally advanced cervical cancer) | II | NCT04884906 |
| Toripalimab and Anlotinib with Paclitaxel, Cisplatin/ Carboplatin | Evaluation of the effectiveness of combination therapy of paclitaxel with cisplatin / carboplatin, toripalimab and anlutinib as firsy line treatment in the target group | PD-L1 | IA-IVA | Patients with recurrent, metastatic or persistant cervical cancer after platinum-based surgery or chemotherapy or both | I | NCT04731038 |
| Pembrolizumab | Evaluation of PD-L1 expression in CIN lesions as a biomarker following treatment; Determine the HPV clearance status | PD-L1 | - | Cervical Intraepithelial Neoplasia (CIN) | II | NCT04712851 |
| Combination of nimotuzumab (IgG1) and radiotherapy | Evaluation of the effectiveness of combination therapy with nimotosumab plus radiotherapy in inhibiting the expression of anti-epidermal growth factor receptor | EGFR | - | Recurrent or advanced cervical cancer | II | NCT04664244 |
| NP137 with Pembrolizumab and/ or chemotherapy | Evaluation of the effect and clinical activity of NP137 following combination with pembrolizumab or chemotherapy | Netrin-1  /  PD-1 | - | Advanced or metastatic gynecological cancers (Endometrial carcinoma or cervical carcinoma) | I/II | NCT04652076 |
| Toripalimab (IgG4) plus Chidamide | Assessment of the efficacy and clinical activity of toripalimab following combination with chidamide | PD-1 | - | Recurrent metastatic or persistent, cervical cancer cases | I/II | NCT04651127 |
| Cemiplimab in combination with ISA101b vaccine | Determinate of clinical actuality of cemiplimab + ISA101b as the first line of treatment, after the progression of malignancy following chemotherapy | PD-1 | - | Recurrent or metastatic HPV16 cervical cancer | II | NCT04646005 |
| Pembrolizumab with olaparib | Pembrolizumab in combination with Olaparib, in cancer progression cases after platinum-based chemotherapy. | PD-L1 | - | Recurrent or metastatic cervical cancer | II | NCT04641728 |
| Sintilimab plus  IBI310 or Placebo | Estimation of clinical actuality and efficacy of sintilimab in combination with IBI310 or Placebo for second-line treatment | PD-1  CTLA-4 | - | Advanced cervical cancer patient who have been failed or cannot tolerate platinum-based chemotherapy in the first line of treatment or higher | II | NCT04590599 |
| Camrelizumab with Capecitabine | Evaluation the efficiency of camrelizumb plus metronomic capecitabine | PD-L1 | - | Advanced cervical cancer | I | NCT04508686 |
| Pembrolizumab  with  Olaparib | Investigation the efficiency of pembrolizumab plus  olaparib | PD-1 | - | Advanced cervical cancer or recurrent after standard chemotherapy | II | NCT04483544 |
| Ociperlimab  and   Tislelizumab | To evaluate the efficacy and safety of tislelizumab combined with or without ociperlimab (BGB-A1217) | PD-1  and  TIGIT | - | Recurrent  Or  Metastatic | II | NCT04693234 |
| Combination therapy of atezolizumab plus VB10.16 vaccination | Determination of efficacy and clinical activity of multiple doses of VB10.16 in combination with atezolizumab and evaluation of HPV16 E6/E7 specific cellular immune responses | PD-L1  and specific cellular immune response versus E6/E7 oncogenes | - | Advanced or recurrent cervical cancer cases with HPV16 positive | II | NCT04405349 |
| Tiragolumab  and  Atezolizumab | To evaluate the efficacy and safety of tiragilumab in combination with atezolizumab | PD-L1 | - | Recurrent  Or  Metastatic | II | NCT04300647 |
| Pembrolizumab  And  Vibostolimab | Pembrolizumab/vibostolimab co-formulation is superior to pembrolizumab alone in terms of objective response rate or progression-free survival in participants with cervical cancer. | PD-L1 | - | Uterine cervical neoplasms | II | NCT05007106 |
